# The Effects of Edaphic Factors on Riparian Plants in the Middle and Lower Reaches of the Hanjiang River, China

**DOI:** 10.3390/plants11040531

**Published:** 2022-02-16

**Authors:** Jiao Yang, Enhua Li, Rui Zhou, Ying Xia, Chao Yang, Yingying Zhang

**Affiliations:** 1 Key Laboratory for Environment and Disaster Monitoring and Evaluation of Hubei Province, Innovation Academy for Precision Measurement Science and Technology, Chinese Academy of Sciences, Wuhan 430077, China; yangjiao05216@163.com (J.Y.); zhourui1@apm.ac.cn (R.Z.); xiaying@apm.ac.cn (Y.X.); cyang@whigg.ac.cn (C.Y.); 2 University of Chinese Academy of Sciences, Beijing 100049, China; 3 School of Geography and Tourism, Zhengzhou Normal University, Zhengzhou 450044, China; zhangyywhigg@163.com

**Keywords:** riparian plant, hydrophilicity, life history type, diversity, soil, RDA, Hanjiang River

## Abstract

It is important to understand the interactions between soil and plant in riparian zones to ensure ecosystem function. The effects of edaphic factors on plant composition and species diversity were investigated in the middle and lower reaches of the Hanjiang River (MLHR), China. A total of 154 species of herbs were recorded, and vegetation was divided into 32 clusters according to a two-way indicator species analysis (TWINSPAN). *Cynodon dactylon* and *Paspalum distichum* were the most common clusters, accounting for 22.7% and 12.5% of total samples, respectively. Hydric and mesic species were dominant in the first and second transects, with mesophytes dominating the third and fourth transects. First transects situated were mainly composed of perennials and annuals, respectively. Perennials in second transects were present in 83% of sites, and perennials in the third and fourth transects were present in 86% of the sites. Margalef richness index (Dma), Simpson dominance diversity index (D), Shannon–Wiener diversity index (H) and Pielou evenness index (Jsw) were higher in the first transects in some sites. The redundancy analysis (RDA) results indicated that soil moisture content was the dominant factor affecting hydrophilic vegetation types, and total nitrogen (TN) and soil organic matter (SOM) were the most critical factors affecting plant life history types and species diversities in this area.

## 1. Introduction

The riparian ecosystem is an important primary producer in a river ecosystem as ecotones between terrestrial and aquatic ecosystems [1]. The riparian ecosystem includes landscapes above high watermarks, consisting of channels between low- and high-water levels where vegetation may be affected by rising water levels or flooding, and by the capacity of the soil to retain water [2]. Vegetation in the riparian ecosystem also provides a significant barrier reducing erosion and non-point pollutants [3]. The quality of a river ecosystem environment can also be reflected by the types of plants, species composition and the spatial variation pattern of species diversity along an environmental gradient [4,5].

Studies examining vegetation types, diversity, and environmental impact factors form the basis of ecology, and they are the core of conservation biology [6]. Compared with rivers in a natural environment, rivers in plain areas affected by anthropogenic activities form a close relationship with outside influences. Today, the majority of river riparian belts has been fragmented by anthropogenic activities, for example by the construction of embankments to prevent natural disasters such as floods [7,8], the construction of dams, and other water conservancy projects [9], as well as the expansion of urban and cultivated land areas [10]. As ecosystems in these riparian zones are very fragile, it is therefore important to study the interrelationship between plants and the environment in this area [11]. Previous studies have shown that species composition and diversity distribution patterns of river riparian zones are controlled by factors such as climate, topography, hydrology, soil, and anthropogenic activities [2,6,7,12]. Among the different environmental factors, soil is the basis of habitat heterogeneity, climate, and biology, controlling the ecological process of the ecosystem, and playing a key role in community distribution and characteristics [6]. A decrease in soil moisture variables has been recorded to result in seedling mortality for some species and nutrient absorption by plants [13,14], leading to different adaptation types of plants along the water gradient [15]. In addition, the growth of plants that have adapted to these alluvial soil conditions has benefited from the regular supply of nutrients [16,17]. On a regional scale, soil factors determining plant growth are affected by climate, human activities, and other edaphic factors. It is of great significance, therefore, to analyze the relationship between plants and soil factors.

The middle and lower reaches of the Hanjiang River (MLHR), located to the north of the Jianghan Plain, are regions with frequent anthropogenic activities in China. With the construction of the middle route of the south-to-north water transfer project, and water transfer from the Yangtze River to the Hanjiang River, water conservation in the MLHR has become a key area of environmental research [18,19], with studies examining plant protection in the riparian zones being important to ensure ecosystem function. Currently, studies on plants in the MLHR have predominantly focused on community characteristics, species composition, and influencing factors of aquatic flora [20,21]; studies examining interactions between vegetation and the environment in the MLHR riparian zones are lacking. It is therefore important to examine riparian plants in the MLHR for ecological protection and restoration, as well as for the ecological operation of reservoirs and river management. By using the results from our study, we aim to answer the following two questions: (1) Is there a difference in vegetation stratification along the elevation of the MLHR? and (2) What are the key factors contributing to these differences?

## 2. Results

### 2.1. Community Composition

In the field survey, it was found that the plants showed obvious frontal zonal distribution along the water edge to the height; based on the characteristics of plant zonal distribution, each sampling site was divided into 2–4 transects. A total of 154 herb species (including outside the sites), belonging to 128 genera and 55 families, were recorded over the two study periods (Table S1). There were 5 species of ferns in 5 genera and 5 families, and 149 species of seed plants in 123 genera and 50 families; however, gymnosperms were not recorded. Among the seed plants, 43 species were monocotyledons and 106 species were dicotyledons. Between the two study periods, little difference in plant species and composition was recorded, with 133 species in 2018 and 131 species in 2019. The largest family was Poaceae with 24 species (accounting for 15.58% of the total species), followed by Asteraceae (20 species, 12.99%), Cyperaceae (15 species, 9.74%), and Polygonaceae (6 species, 3.90%). The largest genera were *Potamogeton* and *Polygonum* (5 species each), followed by *Scirpus*, *Cyperus,* and *Artemisia* (3 species each). There were three invasive alien species (*Eichhornia crassipes, Alternanthera philoxeroides,* and *Trifolium repens*).

**Figure 1:**
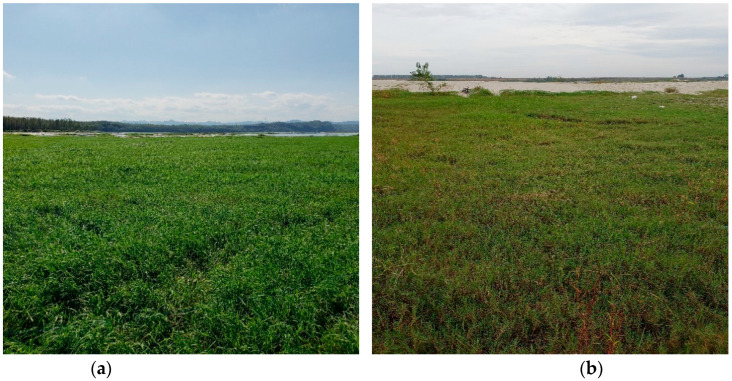
(**a**) *Paspalum distichum* community (the first transect of Shanbiancun (SBC), September 28, 2018); (**b**) *Cynodon dactylon* community (the second transect of Zekou (ZK), 1 October 2018).

Results for two-way indicator species analysis (TWINSPAN) in the study sites recorded plants in the middle and lower reaches of the Hanjiang River (MLHR) to be divided into 32 clusters (Table S2). The majority of community types were *Cynodon dactylon* and *Paspalum distichum* communities, accounting for 22.7% and 12.5% of the total number of transects, respectively (Figure 1). In the first transects, dominant community species near the water edge included *C. dactylon, P. distichum*, *Hemarthria altissima*, *Phalaris arundinacea*, *Leersia hexandra,* and *Typha angustifolia*. Dominant species in the second transects included *C. dactylon, Phragmites australis, Triarrhena lutarioriparia*, *Scirpus triqueter*, and *P. distichum*. Dominant species in the third transects included *C. dactylon, Imperata cylindrica*, *Bromus japonicus*, *P. distichum,* and *Artemisia argyi*. The fourth transects, located the furthest away from the water’s edge, was dominated by *I. cylindrica*. Dominant species in the first and the second transects were mainly perennial hydric plants and mesic plants; perennial mesophytes mainly dominated the third and the fourth transects.

### 2.2. Community Hydrophilicity and Life History Characteristics

**Figure 2:**
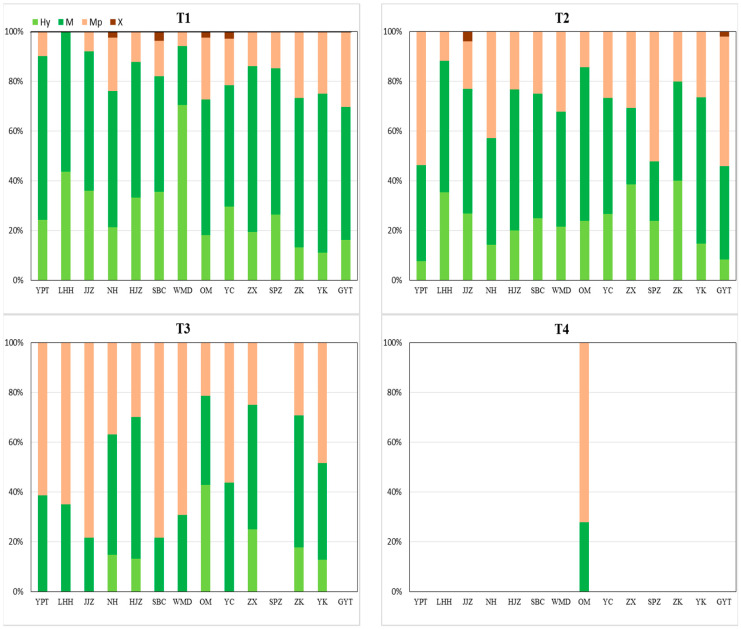
Plant hydrophilic types for four transects in each sampling site in the MLHR, China. Hy, hydric species; M, mesic species; Mp, mesophytes; and X, xerophytes. T1, 2, 3, and 4 represent the first, second, third, and fourth transect, respectively. YPT, Yangpitan; LHH, Lihuahu; JJZ, Jiangjiazhou; NH, Nanhu; HJZ, Hujiazhou; SBC, Shanbiancun; WMD, Wumingdao; OM, Oumiao; YC, Yicheng; ZX, Zhongxiang; SPZ, Shipaizhen; ZK, Zekou; YK, Yuekou; GYT, Guanyintang.

**Figure 3:**
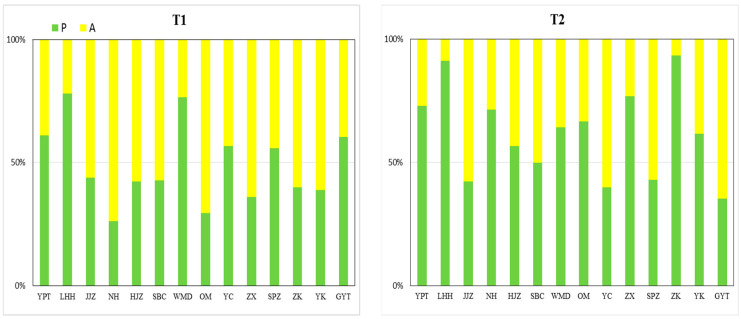

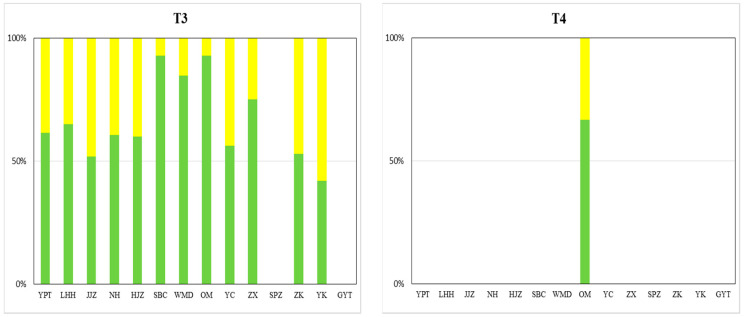
Plant life history types for four transects in each sampling site in the MLHR. P, perennials; A, annuals. T1, 2, 3, and 4 represent the first, second, third, and fourth transect, respectively. YPT, Yangpitan; LHH, Lihuahu; JJZ, Jiangjiazhou; NH, Nanhu; HJZ, Hujiazhou; SBC, Shanbiancun; WMD, Wumingdao; OM, Oumiao; YC, Yicheng; ZX, Zhongxiang; SPZ, Shipaizhen; ZK, Zekou; YK, Yuekou; GYT, Guanyintang.

Results for the percentage of hydrophilic (Figure 2) and life history types (Figure 3) in each transect indicted that the proportion of hydric species and mesic species in the first transects was higher than in other transects. The proportion of hydric species and mesic species in the second transects was higher than mesophytes in the majority of sites; mesophytes recorded a higher proportion in about half of the sites in the third transects and they were dominant in the fourth transects. Xerophytes were only recorded in the first and second transects in sites Nanhu (NH), Jiangjiazhou (JJZ), Shanbiancun (SBC), Oumiao (OM), Yicheng (YC), and Guanyintang (GYT); xerophytes were not recorded in any other site.

In terms of the life history types of plants, perennials were dominant in half of the sites in the first transects, predominantly in beach sites. Annuals in the first transects were also dominant in half of the sites, predominantly in central island sites. The proportion of perennials in the majority of second transects was significantly higher than annuals, and, apart from site Yuekou (YK), perennials dominated the third and fourth transects.

The number of species of hydrophilic and life history types at each transect (Table 1) indicated that hydric species and mesic species significantly decreased from T1 to T4 whilst mesophytes and xerophytes significantly increased. Results for annuals recorded a significant increase from the first transect to the third transect, and a significant decrease from the third to the fourth transect. Results for perennials recorded an opposite trend. In the fourth transect, no significant difference between the number of annuals and perennials was recorded.

### 2.3. Species Diversity Characteristics

**Figure 4:**
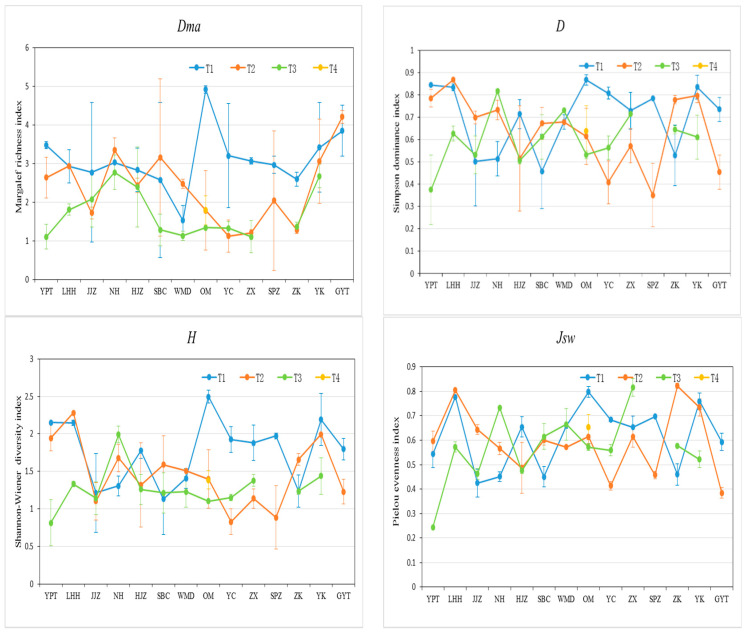
The diversity indices of different transects in the sampling sites in the MLHR, China. Dma, Margalef richness index; D, Simpson dominance diversity index; H, Shannon–Wiener diversity index; and Jsw, Pielou evenness index. T1, 2, 3, and 4 represent the first, second, third, and fourth transect, respectively. YPT, Yangpitan; LHH, Lihuahu; JJZ, Jiangjiazhou; NH, Nanhu; HJZ, Hujiazhou; SBC, Shanbiancun; WMD, Wumingdao; OM, Oumiao; YC, Yicheng; ZX, Zhongxiang; SPZ, Shipaizhen; ZK, Zekou; YK, Yuekou; GYT, Guanyintang.

Variation ranges for the Margalef richness index (Dma), the Simpson dominance diversity index (D), the Shannon–Wiener diversity index (H), and the Pielou evenness index (Jsw) were 1.10–4.91 (2.41 average), 0.35–0.87 (0.65 average), 0.42–2.49 (1.41 mean value), and 0.24–0.82 (0.59 mean value), respectively (Figure 4). Dma, D, H, and Jsw in the first transects were higher than the others, with about 57%, 64%, 57%, and 50% for all sites, respectively.

### 2.4. RDA Ordination and Distribution

**Figure 5:**
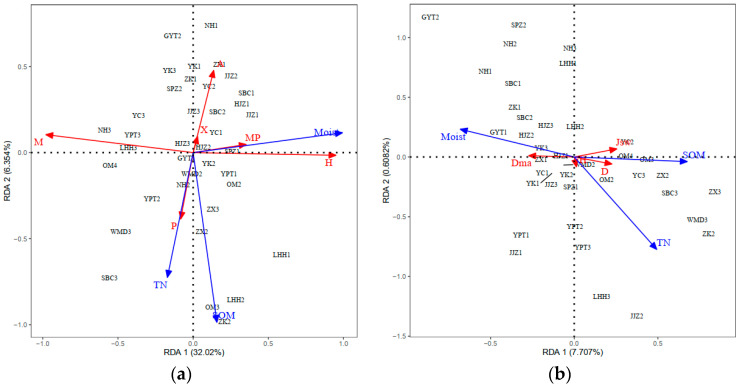
RDA of edaphic factors and species hydrophilic, life history, and species diversity in the MLHR. (**a**) RDA ordination diagram of the edaphic factors and the vegetation types; (**b**) RDA ordination diagram of the edaphic factors and the diversity indices of different transects. Based on the analysis of three sequence diagrams, the long arrow represents the edaphic factor, and the length of the arrow indicates its effect on plant size. Species and edaphic factors represented the cosine value of the relevance of the plant and edaphic factors. P, perennials; A, annuals. Dma, Margalef richness index; D, Simpson dominance diversity index; H, Shannon–Wiener diversity index; Jsw, Pielou evenness index. Hy, hydric species; M, mesic species; Mp, mesophytes; X, xerophytes. TN, total nitrogen; Moist, soil moisture; SOM, soil organic matter. YPT, Yangpitan; LHH, Lihuahu; JJZ, Jiangjiazhou; NH, Nanhu; HJZ, Hujiazhou; SBC, Shanbiancun; WMD, Wumingdao; OM, Oumiao; YC, Yicheng; ZX, Zhongxiang; SPZ, Shipaizhen; ZK, Zekou; YK, Yuekou; GYT, Guanyintang.

Redundancy analysis (RDA) on the indicators of plant community structure (hydrophilic, life history types, and species diversities) and all five environmental variables explained 42.29% of the total variances (Table 2). In order to simplify the model, edaphic factors were selected forward (*p* < 0.05, random displacement test). As soil moisture, soil organic matter (SOM) and total nitrogen (TN) explained 37.25% of the total variances (Table 2), these factors were retained. Species hydrophilicity, life history, and diversities were significantly correlated with soil moisture, SOM, and TN contents, with soil moisture strongly influencing the species hydrophilic types (Figure 5). Although hydric species (Hy) and mesic species (M) were positively correlated with soil moisture (Moist), mesophytes (Mp) were negatively correlated with soil moisture; although, the correlation between xerophytes (X) and soil moisture was not strong, it was recorded to be positive. TN and SOM contents had relatively little effect on species hydrophilic types in the study area. For species life history types, TN and SOM content recorded a greater influence than soil moisture. Perennials were significantly positively correlated with TN and SOM and annuals were negatively correlated. Compared with soil moisture, TN and SOM content had a strong correlation with D, H, and Jsw. TN and SOM content was positively correlated with D, H, and Jsw.

## 3. Discussion

A total of 154 herbaceous vascular plants species of wild plants were recorded in the riparian zones of the middle and lower reaches of the Hanjiang River (MLHR), China. Species were mainly from the families, Poaceae, Asteraceae, Cyperaceae, and Polygonaceae, and they were divided into 32 communities, with *Cynodon dactylon* and *Paspalum distichum* communities being the main groups. This result was basically consistent with vegetation types recorded in surrounding rivers and lakes, such as in Danjiangkou reservoir and the Yangtze River [22,23]. The distribution of different plant community types was shown by Bornman et al. [24] to present obvious belt distribution patterns along a moisture gradient in the Olifants Estuary, South Africa, and Flanagan et al. [25] recorded different plants to be distributed in patches along a hydrological gradient in Virginia and North Carolina, USA. Our results indicate that the landscape of plant communities in the MLHR was obviously stratified, and most areas could be divided into three transects, recording a zonal distribution with elevation.

Redundancy analysis (RDA) results showed that soil moisture strongly influenced the species hydrophilic types. Hydrophilicity of plants is the most direct manifestation of their adaptation to the environment [26]. Generally, hydric and mesic species have certain flood resistance abilities, with the majority having both vegetative propagation and seed reproduction capabilities, enabling them to quickly germinate after flood events [27]. Notably, gramineous plants such as *P. distichum*, *C. dactylon*, and *Hemarthria altissima* can rapidly grow using their stolons after water levels subside, forming dominant populations in areas with frequent water level changes [28,29]. Soil moisture content was positively correlated with hydric and mesic species. Specifically, the first transects were typically closer to the water, thereby having higher soil moisture contents, and flood events lasted longer than at the second and third transects. The third and fourth transects were only flooded during the flood season, or not flooded at all. When soil moisture content was low, the proportion of mesophytes was high. In the MLHR, from the first to the fourth transects, a gradual decrease in soil moisture resulted in a good transition of vegetation types, following the relationship between plant hydrophilic types and the water environment [30].

In addition, it has been shown by Osawa et al. [31] and Greet et al. [32] that river flow plays an important role in seed dispersion. Compared with the third and the fourth transects, the first and the second transects are more frequently affected by river water levels and plant seeds are more likely to be deposited in the upper water body when water levels drop [33], this, therefore, being the main reason for the discovery of xerophytes only in the first and second transects.

RDA results showed that total nitrogen (TN) and soil organic matter (SOM) have a significant influence on plant life history types, and TN and SOM content was positively correlated with perennial distribution and negatively correlated with annual distribution. Life history type is one of the most useful characteristics for describing a community environment. Generally, perennial plants are suitable for growth in a more stable environment, while annuals, due to their rapid growth rate, can adapt to a more changeable environment [26,34]. Higher TN and SOM contents provide a more stable and superior growth environment for perennials, which can obtain more light and other resources before the germination of annuals, thereby limiting the growth of annuals [35]. On the other hand, plants and the environment are an interdependent complete system. Plants can obtain TN and SOM from the soil as well as being an important source of TN and SOM [36]. Compared with annuals, perennials have a more developed root system and higher overland morphology, which can intercept nutrients brought by rainfall runoff and from floods (including a large number of litter), thereby improving their soil nutrient quality [37].

Findings by Wang et al. [38] highlighted that the Hanjiang River basin has a high use of agricultural chemical fertilizers, severe pollution from non-point sources, and an abundant source of nutrients. Soil nutrients associated with rainfall runoff are relatively high, and they initially pass through the fourth and third transects, resulting in the proportion of perennials in these transects being higher than that of annuals. The proportion of perennials in the first transects differed at different sites. In the riparian zones, perennials were dominant in the first transects where rich soil ensured their growth [38]. However, the proportion of annuals in the first transects was greater in the central island, a result that may be due to the lack of anthropogenic activities in these areas, and the lack of upper rich soil sources. Furthermore, TN, SOM, and plants could be quickly washed away due to serious levels of water erosion [14], making it difficult for perennials to establish long-term colonization.

Species diversity can directly or indirectly reflect environmental types, stability, and habitat differences [39]. In general, species diversity was low, reflecting a simple community structure [6]. In the MLHR, species diversity was positively correlated with TN and SOM contents, and the effect of soil moisture content was relatively small. Areas with high species diversity were mainly concentrated in the first and second transects. Although differences between the majority of transects were not significant, differences among various indicators of diversity were evident, which fully reflected the complexity of marginal zone habitats in the MLHR.

Li et al. [6] recorded that TN is one of the most important edaphic factors affecting species diversity in the Heihe River Basin, and SOM has been previously reported to be the most important limiting factor affecting species diversity in river fringe areas in arid regions [11,40]. Previous studies have also suggested that appropriate increases in TN and SOM contribute to the improvement of species diversity [41]. RDA results indicated that TN and SOM content had the greatest influence on species diversity indices (H, D, and Jsw), and they were positively correlated with the indices. Although a close relationship exists between species diversity and soil, key factors influencing plant composition differ between rivers [6,25].

However, the formation and maintenance of plant communities in the riparian zones is the result of synergy between edaphic factors and long-term accumulation [42,43]. In particular, direct modification of the natural environment by anthropogenic activities, such as the increasing number of cascade dams, will result in a reduction in riparian area and a reduction in living space for emergent water plants and moist plants in this zone [44,45]. As the study only indirectly discussed the effect of soil on plants in the riparian zones, future investigations will combine other factors, such as the influence of anthropogenic activities, with the composition of plants in the MLHR to identify the mechanism of community maintenance and succession.

## 4. Materials and Methods

### 4.1. Study Area

**Figure 6:**
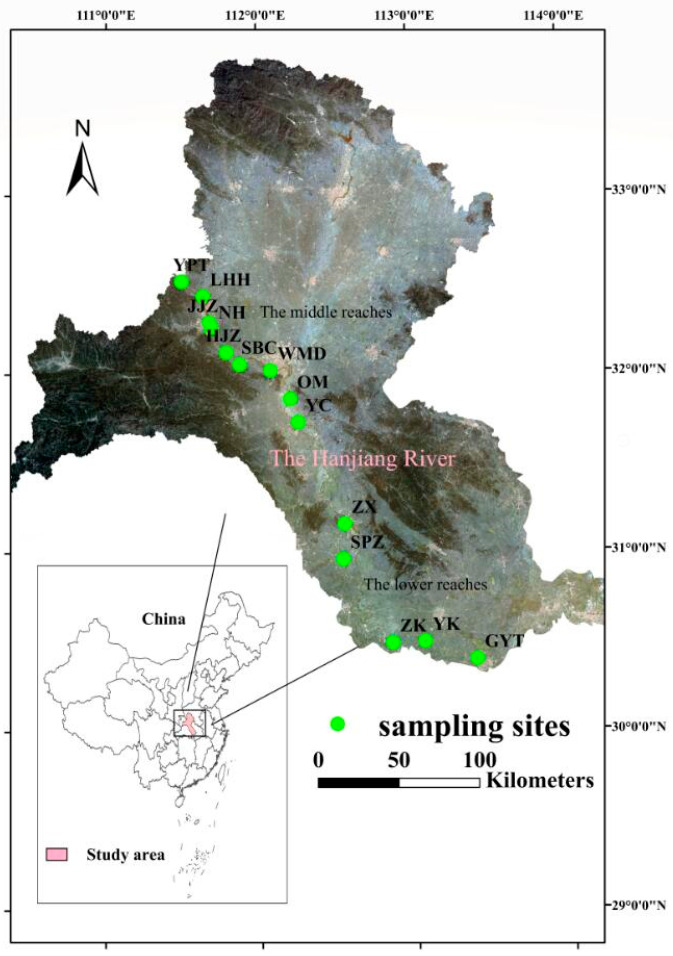
Sampling site locations in the MLHR. The sampling sites were named after the abbreviation of the local place names: YPT, Yangpitan (B); LHH, Lihuahu (B); JJZ, Jiangjiazhou (I); NH, Nanhu (I); HJZ, Hujiazhou (B); SBC, Shanbiancun (I); WMD, Wumingdao (I); OM, Oumiao (I); YC, Yicheng (B); ZX, Zhongxiang (I); SPZ, Shipaizhen (I); ZK, Zekou (B); YK, Yuekou (B); GYT, Guanyintang (B). B in parentheses indicates a beach location and I in parentheses indicates a central island.

The Hanjiang River, located between 106–114° E and 30–34° N, with a total length of 1570 km and a basin area of 170,400 km^2^, is the longest tributary of the Yangtze River rising south of Qinling mountain, China. The basin area accounts for 8.8% of the Yangtze River basin area [46]. The terrain of the basin is high in the west and low in the east, gradually descending from the middle and low mountains in the west to the hilly plain area to the east. Our study area was located in the middle and lower reaches of the Hanjiang River (MLHR), having a total length of 652 km and a basin area of 64,000 km^2^ (Figure 6) [47]. The study site is situated below the Danjiangkou Reservoir (32°33′25″ N, 111°29′31″ E). The basin is situated in the East Asian subtropical monsoon climate zone, with an average temperature of 15–16.5 °C, a frost-free period of more than 230–260 days, and mean precipitation of 700–1300 mm [18]. The middle reaches of the river valley are wide, and during the dry season is 0.3–0.4 km wide and the flood season is 2–3 km wide. The downstream area is slow and the channel narrates, being less than 0.2 km wide near the estuary [48].

### 4.2. Plant Data Collection

Vegetation surveys focusing on riparian herbs were undertaken in the MLHR in the autumn of 2018 (in September) and the summer of 2019 (in June). Because herbaceous plants were the most dominant vegetation in the actual field survey, our research just included herbaceous vascular plants (ferns and seed plants). Fourteen sampling sites were established in 14 typical areas, extending from site Yangpitan (YPT), next to the dam of the Danjiangkou Reservoir, to site Guanyintang (GYT), the last natural riparian area in the lower reaches of the Hanjiang River (Figure 6). Due to serious human interference, sites were not present in the confluence of the Hanjiang River and the Yangtze River.

Each sampling site was divided into 2–4 transects, where species composition, community structure, and community elevation were relatively consistent in each transect. Three to six 1 m × 1 m quadrats were situated parallel to the water surface in each transect. There were 145 and 135 quadrats in September (2018) and June (2019), respectively. In each quadrat, we recorded the coverage of each species and the total coverage of the quadrat (based on the sum of the crown areas), which were expressed as a percentage, ranging from 0 to 100%. The coverage was determined by visual estimation. The plant height and the number of individuals were also recorded. For tufty herbs, the number of stems was regarded as the number of individuals [26]. The quadrat locations (elevation, longitude, and latitude) were recorded using a GPS receiver (ATK-S82_2013_, Nanjing Hanzhong Surveying Instruments Co., Ltd., Nanjing, China).

### 4.3. Edaphic Parameters

To eliminate the influence of precipitation on soil moisture content, soil moisture for each quadrat was recorded at the thickness of 0–10 cm using a soil HD2 hygrometer (IMKO Micromodultechnik, Germany) after a week without precipitation [6]. About 500 g of soil was collected from each quadrat at the 0–10 cm depth, and all soil samples were mixed from the same transect into one soil sample. Soil samples were naturally air-dried after removing debris and screened using a 0.088 mm sieve for fine and coarse soil components [26]. Total nitrogen (TN) and total phosphorus (TP) were measured using the overflow acid potassium digestion method [49]. Total organic matter (SOM) was determined by computing weight loss after ignition at 600 °C [1].

### 4.4. Data Analysis

Plant species were divided into four hydrophilic types based on their adaptability (hydric species, mesic species, mesophytes, and xeric species). Similarly, according to plant life history, species were divided into two major categories (annual and perennial species). Plants were divided into hydrophilic and life history types by reference to the flora of China (http://www.iplant.cn/foc/, accessed on 10 January 2022). Species importance value (IV) and diversity indices in each transect were calculated as [6,50]:

IV = (Relative density + Relative height + Relative coverage)/3(1)

Margalef richness index (Dma):

(2) $$Dma=\left(N_{0}-1\right)/\ln N$$

Simpson dominance diversity index (D):

(3) $$D=1-\overset{S}{\underset{i=1}{\sum }}{\left(P_{i}\right)}^{2}$$

Shannon–Wiener diversity index (H):

(4) $$H=-\overset{S}{\underset{i=1}{\sum }}P_{i}\ln\left(P_{i}\right)$$

Pielou evenness index (Jsw):

(5) Jsw=H/lnS

where *S* is the number of species in the quadrat; *N* is the total number of individuals of all species in the quadrat; *P_i_* = *N_i_*/*N*_0_, *N_i_* is the number of species *i*; and *N*_0_ is the total number of all species.

The floristic data matrix was then classified into vegetation clusters by two-way indicator species analysis (TWINSPAN) using the default settings of the computer software PC-ORD (version 5) [1].

Hydrophilicity, life history characteristics, diversity indices, and soil properties of the different transects were compared using analysis of variance (ANOVA) and Duncan’s test (*p* < 0.01 or *p* < 0.05). Data pretreatment and analysis were completed using Excel 2010 [26]. Descriptive statistical parameters and significance tests were conducted using SPSS (version 18.0, IBM SPSS Statistics, Chicago, IL, USA).

In order to explore differences in hydrophilic and life history types between different sample site transects, the percentage of hydrophilic and life history types in each transect in the total number of species was used to characterize the composition in different transects.

Detrended correspondence analysis (DCA) was used to analyze the correlation to choose linear- or unimodal-based methods between edaphic factors and species hydrophilic, life history types, and species diversity by DCA axis 1. DCA in the data set (1.2) showed that the gradient length of axis 1 was less than 3.0, indicating that most species exhibit a linear response to potential environmental changes, therefore suggesting that the use of a linear multivariable approach was reasonable. In our study, key edaphic factors were identified using RDA analysis.

Collinearity of the data was tested by measuring variance inflation factors (VIF) of variables; test results showed that all VIF values of environmental variables were less than 10, and there was no collinearity. Environmental factor variables were pre-selected forward to improve the quality of the model. In the process of RDA analysis, the Hellinger method was adopted to transform plant data, and log10 transformation was applied to all environmental factor data. Both the DCA and RDA analyses were undertaken using R (version 3.6.1, R Foundation for Statistical Computing, Vienna, Austria, http://www.R-project.org/ accessed on 2 December 2021).

## 5. Conclusions

Results from the investigation demonstrated that soil moisture, Total nitrogen (TN), and soil organic matter (SOM) contents were the main factors influencing plant hydrophilicity, life history types, and species diversities in the middle and lower reaches of the Hanjiang River (MLHR). Soil moisture content was the dominant factor influencing plant community hydrophilic groups, with higher soil moisture contents coinciding with higher proportions of hydric and mesic species. TN and SOM were the only two statistically significant factors determining plant life history types and species diversity in the study area. The proportion of perennials and species diversities was relatively higher in the riparian environment where TN and SOM contents were higher, and soil moisture had less influence. Therefore, when managing the MLHR environment and other similar riparian zones in the world, it is important to consider soil moisture, TN, and SOM to ensure species diversity and ecosystem function.

## Figures and Tables

**Table 1:** Plant hydrophilicity and life history characteristics of the four transects in the MLHR, China.

Index	Transect			
	T1	T2	T3	T4
**Hydrophilicity group**				
Hydric species	19	12	8	2
Mesic species	64	58	37	8
Mesophytes	27	36	40	14
Xerophytes	1	1	1	0
**Life-history**				
Perennial species	49	46	36	12
Annual species	62	61	50	12

**Table 2:** Regression coefficient (canonical characteristic coefficient) and sorting summary between edaphic factors and sorting extraction. TN, total nitrogen; TP, total phosphorus; SOM, soil organic matter.

	Axis 1	Axis 2	Axis 3	Axis 4	Random Permutation Test	
					R^2^	*p*
Altitude	0.011	0.108	−0.068	−0.122	0.084	0.215
Soil moisture	0.560	−0.060	−0.199	−0.082	0.539	0.001 ***
TN	−0.001	−0.049	−0.570	−0.107	0.1673	0.027 *
TP	−0.347	1.207	0.381	−1.514	0.0071	0.888
SOM	0.012	0.525	0.467	0.366	0.445	0.001 ***
Eigenvalues	0.018	0.006	0.001	0.0001		
Species-environment correlations	0.738	0.581	0.431	0.314		
Significance test for all axes	F = 4.6202 *p =* 0.001 ***					

* Significant at *p* < 0.05; *** Significant at *p* < 0.001.

## Data Availability

Data sharing not applicable.

## Supplementary Materials

The following supporting information can be downloaded at: https://www.mdpi.com/article/10.3390/plants11040531/s1, Table S1: The list of herb species over the two study periods in the middle and lower reaches of the Hanjiang River; Table S2: The community types over the two study periods in the middle and lower reaches of the Hanjiang River.
