# Supplementary material for: The Effects of Edaphic Factors on Riparian Plants in the Middle and Lower Reaches of the Hanjiang River, China
Source: Plants (Basel). 2022 Feb 16;11(4):531. doi: 10.3390/plants11040531 (PMC8874981; doi:10.3390/plants11040531)
Supplement: Supplementary file 1 [file plants-11-00531-s001.zip › plants-1584447-supplementary.pdf]

Supplementary Materials

Table S1. The list of herb species over the two study periods in the middle and lower reaches of the Hanjiang River.

No.	Families	Genera	Species	Hy	M	Mp	X	P	A
1	Pteridaceae	*Pteris*	*Pteris multifida* Poir.		√			√	
2	Eguisetaceae	*Equisetum*	*Equisetum ramosissimum* Desf.		√			√	
3	Nephrolepidaceae	*Nephrolepis*	*Nephrolepis auriculata* (L.) Trimen		√			√	
4	Salviniaceae	*Salvinia*	*Salvinia natans* (L.) All.	√				√	
5	Azollaceae	*Azolla*	*Azolla pinnata* subsp. asiatica R. M. K. Saunders & K. Fowler	√				√	
6	Typhaceae	*Typha*	*Typha angustifolia* Linn.	√				√	
7	Potamogetonaceae	*Potamogeton*	*Potamogeton crispus* Linn.	√				√	
8			*Potamogeton maackianus* A. Bennett	√				√	
9			*Potamogeton wrightii* Morong	√				√	
10			*Potamogeton pectinatus* Linn.	√				√	
11			*Potamogeton perfoliatus* Linn.	√				√	
12	Hydrocharitaceae	*Elodea*	*Elodea canadensis*	√				√	
13		*Hydrilla*	*Hydrilla verticillata* (L. f.) Royle	√				√	
14		*Hydrocharis*	*Hydrocharis dubia* (Bl.) Backer	√				√	
15		*Vallisneria*	*Vallisneria natans* (Lour.) Hara	√				√	
16	Poaceae	*Alopecurus*	*Alopecurus aequalis* Sobol.		√				√
17		*Arthraxon*	*Arthraxon hispidus*		√				√
18		*Bromus*	*Bromus japonicus* Thunb. ex Murr.			√			√
19		*Cynodon*	*Cynodon dactylon* (L.) Pers.			√		√	
20		*Digitaria*	*Digitaria sanguinalis* (L.) Scop.			√			√
21		*Echinochloa*	*Echinochloa caudata* Roshev.		√				√
22			*Echinochloa colonum* (L.) Link		√				√
23		*Eleusine*	*Eleusine indica* (L.) Gaertn.				√		√
24		*Hemarthria*	*Hemarthria sibirica* (Gandoger) Ohwi		√			√	
25		*Imperata*	*Imperata cylindrica* (Linn.) Beauv.				√	√	
26		*Isachne*	*Isachne globosa* (Thunb.) Kuntze			√		√	
27		*Leersia*	*Leersia hexandra* Swartz.		√			√	
28		*Lolium*	*Lolium perenne* L.		√			√	
29		*Milium*	*Milium effusum* L.		√			√	
30		*Paspalum*	*Paspalum distichum* (Michx.) Scribn.		√			√	
31		*Pennisetum*	*Pennisetum alopecuroides* (L.) Spreng.			√		√	
32		*Phalaris*	*Phalaris arundinacea* Linn.		√			√	
33		*Phragmites*	*Phragmites australis* (Cav.) Trin. ex Steud.		√			√	
34		*Polypogon*	*Polypogon fugax* Nees ex Steud.		√				√
35		*Saccharum*	*Saccharum arundinaceum* Retz.				√	√	
36		*Setaria*	*Setaria viridis* (L.) Beauv.				√		√
37		*Triarrhena*	*Triarrhena lutarioriparia* L. Liou var. gongchai L.Liu				√	√	
38			*Triarrhena sacchariflora* (Maxim.) Nakai		√			√	
39		*Zizania*	*Zizania latifolia* (Turcz) Hand.-Mazz.	√				√	
40	Cyperaceae	*Pycreus*	*Pycreus sanguinolentus* (Vahl) Nees		√				√

41		*Carex*	*Carex brachyathera* Ohwi			√		√
42			*Carex neurocarpa* Maxim.		√			√
43		*Cyperus*	*Cyperus iria* L.		√			√
44			*Cyperus pilosus* Vahl		√			√
45			*Cyperus rotundus* Linn.		√		√	
46		*Fimbristylis*	*Fimbristylis squarrosa* var. esquarrosa Makino		√			√
47		*Eleocharis*	*Eleocharis dulcis* (N. L. Burman) Trinius ex Henschel		√		√	
48			*Eleocharis yokoscensis* (Franch. et Savat.) Tang & F. T. Wang		√			√
49		*Juncellus*	*Cyperus serotinus* Rottb.		√		√	
50		*Kyllinga*	*Kyllinga brevifolia* Rottb.		√			√
51		*Lipocarpha*	*Lipocarpha chinensis* (Osbeck) Kern		√			√
52		*Scirpus*	*Scirpus planiculmis* Fr. Schmidt		√		√	
53			*Scirpus triangulatus* Roxb.		√		√	
54			*Scirpus triqueter* Linn.		√		√	
55	Araceae	*Acorus*	*Acorus calamus* Linn.		√		√	
56	Lemnaceae	*Lemna*	*Lemna minor* L.	√				√
57	Commelinaceae	*Commelina*	*Commelina communis* Linn.	√				√
58	Pontederiaceae	*Eichhornia*	*Eichhornia crassipes* (Mart.) Solms$^{\triangle}$	√				√
59	Juncaceae	*Juncus*	*Juncus effusus* Linn.		√		√	
60			*Juncus prismatocarpus* R. Br.		√		√	
61	Saururaceae	*Houttuynia*	*Houttuynia cordata* Thunb.		√		√	
62	Moraceae	*Humulus*	*Humulus scandens* (Lour.) Merr.			√	√	
63	Polygonaceae	*Polygonum*	*Polygonum aviculare* L.			√		√
64			*Polygonum criopolitanum* Hance			√		√
65			*Polygonum hydropiper* L.		√			√
66			*Polygonum lapathifolium* L.		√			√
67			*Polygonum perfoliatum* L.			√		√
68		*Rumex*	*Rumex dentatus* L.		√			√
69	Chenopodiaceae	*Chenopodium*	*Chenopodium album* Linn.			√		√
70			*Chenopodium ambrosioides* Linn.			√		√
71	Amaranthaceae	*Achyranthes*	*Achyranthes bidentata* Blume			√		√
72		*Alternanthera*	*Alternanthera philoxeroides* (Mart.) Griseb.$^{\triangle}$		√		√	
73			*Alternanthera sessilis* (Linn.) DC.		√		√	
74		*Amaranthus*	*Ameranthus spinosus*			√		√
75		*Celosia*	*Celosia argentea* L.			√		√
76	Phytolaccaceae	*Phytolacca*	*Phytolacca acinosa* Roxb.			√		√
77	Aizoaceae	*Mollugo*	*Mollugo stricta* L.		√			√
78	Portulacaceae	*Portulaca*	*Portulaca oleracea* L.		√			√
79	caryophyllaceae	*Arenaria*	*Arenaria serpyllifolia* L.		√			√
80		*Myosoton*	*Myosoton aquaticum* (L.) Moench		√			√
81	Ceratophyllaceae	*Ceratophyllum*	*Ceratophyllum demersum* Linn.	√			√	
82	Ranunculaceae	*Clematis*	*Clematis florida* Thunb.		√		√	
83		*Ranunculus*	*Ranunculus sieboldii* Miq.		√			√
84			*Ranunculus sceleratus* Linn.		√			√

85	Cruciferae	*Cardamine*	*Cardamine hirsuta* L.		√				√
86		*Lepidium*	*Lepidium apetalum*		√				√
87		*Rorippa*	*Rorippa globosa* (Turcz.) Hayek		√				√
88			*Rorippa indica* (Linn.) Hiern		√				√
89	Crassulaceae	*Sedum*	*Sedum sarmentosum* Bunge		√			√	
90	Rosaceae	*Duchesnea*	*Duchesnea indica* (Andr.) Focke		√			√	
91		*Potentilla*	*Potentilla discolor* Bge			√			√
92			*Potentilla fulgens* Wall. ex Hook.			√			√
93	Leguminosae	*Glycine*	*Glycine soja* Sieb. et Zucc.				√		√
94		*Melilotus*	*Melilotus officinalis* (L.) Pall.				√		√
95		*Trifolium*	*Trifolium repens*△			√		√	
96		*Vicia*	*Vicia hirsuta* (L.) S. F. Gray			√			√
97			*Vicia sepium*			√			√
98	Oxalidaceae	*Oxalis*	*Oxalis corniculata* Linn.		√			√	
99	Geraniaceae	*Geranium*	*Geranium carolinianum* L.			√			√
100	Euphorbiaceae	*Acalypha*	*Acalypha australis* L.		√				√
101		*Euphorbia*	*Euphorbia humifusa* Willd.		√				√
102	Vitaceae	*Cayratia*	*Cayratia japonica* (Thunb.) Gagnep.			√			√
103	Violaceae	*Viola*	*Viola prioantha* Bunge		√			√	
104	Trapaceae	*Trapa*	*Trapa incisa* Sieb. et Zucc.	√					√
105	Onagraceae	*Ludwigia*	*Ludwigia hyssopifolia* (G. Don) Exell		√				√
106		*Oenothera*	*Oenothera glazioviana* Mich.			√			√
107	Haloragidaceae	*Myriophyllum*	*Myriophyllum spicatum* L.	√				√	
108	Umbelliferae	*Daucus*	*Daucus carota*			√			√
109		*Hydrocotyle*	*Hydrocotyle sibthorpioides* Lam.		√			√	
110		*Oenanthe*	*Oenanthe javanica* (Bl.) DC.		√			√	
111	Primulaceae	*Penthorum*	*Penthorum chinense* Pursh		√			√	
112	Gentianaceae	*Nymphoides*	*Nymphoides peltatum* (Gmel.) O. Kuntze	√				√	
113	Asclepiadaceae	*Metaplexis*	*Metaplexis japonica* (Thunb.) Makino			√			√
114	Convolvulaceae	*Calystegia*	*Calystegia hederacea* Wall.			√			√
115	Boraginaceae	*Trigonotis*	*Trigonotis peduncularis* (Trev.) Benth. ex Baker et Moore		√				√
116	Verbenaceae	*Phyla*	*Phyla nodiflora*		√			√	
117	Labiatae	*Elsholtzia*	*Elsholtzia cypriani* (Pavol.) C. Y. Wu et S. Chow		√			√	
118		*Leonurus*	*Leonurus artemisis* (Lour.) S. Y. Hu		√				√
119		*Salvia*	*Salvia plebeia* R. Br.		√				√
120		*Scutellaria*	*Scutellaria barbata*		√			√	
121		*Stachys*	*Stachys japonica*		√			√	
122	Solanaceae	*Solanum*	*Solanum nigrum* L.			√		√	
123	Scrophulariaceae	*Mazus*	*Mazus japonicus* (Thunb.) O. Kuntze		√				√
124		*Veronica*	*Veronica polita* Fries		√			√	
125			*Veronica undulata* Wall.		√				√
126	Acanthaceae	*Rostellularia*	*Rostellularia procumbens* (L.) Nees		√				√
127	Plantaginaceae	*Plantago*	*Plantago asiatica* L.		√				√
128	Rubiaceae	*Galium*	*Galium aparine* Linn. var. tenerum (Gren. et Godr.) Rchb.		√				√

129		*Paederia*	*Paederia scandens* (Lour.) Merr.			√		√	
130		*Rubia*	*Rubia cordifolia* L.			√		√	
131	Caprifoliaceae	*Sambucus*	*Sambucus javanica* Blume			√		√	
132	Cucurbitaceae	*Actinostemma*	*Actinostemma lobatum*		√				√
133		*Zehneria*	*Zehneria indica* (Lour.) Keraudren			√			√
134	Campanulaceae	*Lobelia*	*Lobelia chinensis* Lour.		√			√	
135	Asteraceae	*Lactuca*	*Lactuca serriola*			√			√
136		*Artemisia*	*Artemisia annua*			√			√
137			*Artemisia argyi* Lévl. et Van.				√		√
138			*Artemisia selengensis* Turcz. ex Bess.		√			√	
139		*Aster*	*Aster subulatus* Michx.		√			√	
140		*Bidens*	*Bidens pilosa* L.		√				√
141		*Carduus*	*Carduus acanthoides* Linn.			√			√
142		*Cirsium*	*Cirsium setosum* (Willd.) MB.			√			√
143		*Conyza Less.*	*Conyza canadensis* (L.) Cronq.				√		√
144		*Dendranthema*	*Dendranthema indicum* (L.) Des Moul.				√	√	
145		*Eclipta*	*Eclipta prostrata* (L.) L.		√				√
146		*Erigeron*	*Erigeron annuus* (L.) Pers.			√			√
147		*Gnaphalium*	*Gnaphalium affine* D. Don.		√				√
148		*Hemistepta*	*Hemistepta lyrata* (Bunge.) Bunge		√				√
149		*Kalimeris*	*Kalimeris indica* (L.) Sch.-Bip.			√		√	
150		*Ixeris*	*Ixeris polycephala* Cass.			√			√
151		*Lactuca*	*Lactuca serriola*			√			√
152		*Sheareria*	*Sheareria nana*		√				√
153		*Siegesbeckia*	*Siegesbeckia orientalis* L.		√				√
154		*Xanthium*	*Xanthium sibiricum* Patrin ex Widder				√		√
Total	55	128	154	20	83	40	11	69	85

△ Invasive alien species. Hy, hydric species; M, mesic species; Mp, mesophytes; X, xerophytes; P, perennials; A, annuals. The identification keys and nomenclature sources for the studied plants referred to the flora of China (http://www.iplant.cn/foc/).

Table S2. The community types over the two study periods in the middle and lower reaches of the Hanjiang River.

		September, 2018				June, 2019			
**No.**	**Sample sites**	**T**	**No. of Species**	**Total coverage (%)**	**Community types**	**T**	**No. of Species**	**Total coverage (%)**	**Community types**
**1**	YPT	b1	9±3	70.20±16.33	*P. distichum, H. sibirica*	b1	15±2	99.67±0.47	*P. arundinacea, H. sibirica*
		b2	6±2	75.67±12.28	*T. lutarioriparia*	b2	9±1	87.33±10.87	*T. lutarioriparia*
		b3	3±0	75.33±14.61	*I. cylindrica*	b3	6±1	94.67±3.09	*I. cylindrica*
**2**	LHH	z1	11±1	95.00±0.00	*L. hexandra*	z1	8±0	95.67±4.03	*T. angustifolia*
		z2	9±1	91.67±2.36	*S. triqueter*	z2	10±2	96.67±4.03	*I. cylindrica*
		z3	5±0	80.00±8.16	*I. cylindrica*	z3	7±1	96.33±2.62	*E. ramosissimum*
**3**	JJZ	b1	3±2	71.67±4.71	*P. distichum*	b1	13±1	99.00±0.00	*P. distichum*
		b2	3±1	88.33±2.36	*P. distichum*	b2	12±2	98.67±0.47	*S. triqueter*
		b3	5±1	88.33±2.36	*P. distichum*	b3	15±2	99.00±0.00	*H. sibthorpioides*
		b4	4±1	56.67±10.27	*C. dactylon, S. arundinaceum*	b4	4±1	54.67±3.30	*C. dactylon*
**4**	NH	z1	9±0	60.00±4.08	*P. fugax, P. distichum*	z1	17±4	79.67±3.68	*P. fugax*
		z2	5±2	50.00±24.83	*C. dactylon*	z2	7±2	93.67±2.62	*C. dactylon*
		z3	7±1	90.00±4.08	*C. dactylon*	z3	9±2	97.33±1.70	*C. dactylon, B. japonicus*
		z4	7±1	73.33±10.27	*I. cylindrica*	/	/	/	/
**5**	HJZ	z1	7±1	25.00±7.07	*P. distichum*	z1	14±2	98.67±0.47	*P. distichum*
		z2	7±2	46.67±13.12	*P. distichum, E. dulcis*	z2	8±2	97.67±1.89	*C. dactylon*
		z3	6±1	88.33±2.36	*C. dactylon*	z3	12±2	99.67±0.47	*C. dactylon*
**6**	SBC	z1	2±1	66.2528.59	*P. distichum*	z1	11±6	78.00±15.94	*P. fugax*
		z2	4±0	63.33±20.14	*C. dactylon, P. distichum*	z2	14±4	97.33±2.36	*P. distichum*
		z3	4±2	91.67±4.71	*C. dactylon*	z3	6±0	79.00±6.98	*A. argyi, P. arundinacea*
**7**	WMD	z1	6±0	93.33±2.36	*T. angustifolia*	z1	7±0	88.00±11.34	*T. angustifolia*
		z2	10±1	88.33±2.36	*L. hexandra*	z2	8±3	98.67±0.47	*E. ramosissimum, I. cylindrica*
		z3	6±2	83.33±6.24	*I. cylindrica*	z3	5±0	95.67±5.44	*A. argyi*
		z4	4±0	81.67±6.24	*I. cylindrica*	/	/	/	/
**8**	OM	z1	12±2	8.33±2.36	*P. arundinacea*	z1	16±1	66.00±2.94	*A. aequalis*
		z2	3±0	93.33±2.36	*P. arundinacea*	z2	11±2	97.33±0.47	*P. arundinacea*
		z3	4±1	88.75±6.50	*C. dactylon*	z3	5±1	43.33±1.25	*B. japonicus*
		z4	6±1	60.00±20.41	*S. arundinaceum, C. dactylon*	z4	4±1	51.33±5.31	*A. argyi, S. arundinaceum*
**9**	YC	b1	6±1	43.33±15.46	*P. distichum, C. dactylon*	b1	15±2	84.00±2.94	*P. distichum*

		b2	3±1	90.00±0.00	*P. distichum*	b2	6±2	95.33±4.50	*C. dactylon, P. distichum*
		b3	5±1	73.33±2.36	*C. dactylon, S. arundinaceum*	b3	4±1	95.00±5.66	*B. japonicus, C. dactylon*
**10**	ZX	z1	10±2	28.33±25.93	*C. dactylon*	z1	14±1	98.33±1.70	*P. distichum*
		z2	3±2	73.33±20.95	*P. australis*	z2	3±2	91.67±2.87	*P. australis*
		z3	3±1	61.67±9.43	*P. australis*	z3	5±1	76.67±15.92	*C. dactylon*
**11**	SPZ	z1	12±1	56.67±2.36	*P. australis, C. dactylon*	z1	10±3	98.33±0.94	*C. dactylon*
		z2	1±0	63.33±23.92	*P. australis*	z2	9±2	83.00±16.93	*P. australis*
**12**	ZK	b1	6±2	46.67±6.24	*C. dactylon, P. distichum*	b1	8±3	58.67±18.80	*C. dactylon*
		b2	5±1	76.67±2.36	*P. australis, P. arundinacea*	b2	5±1	82.67±8.99	*P. arundinacea, C. dactylon*
		b3	4±1	86.67±4.71	*C. dactylon*	b3	8±1	98.33±0.94	*P. distichum, C. dactylon*
		/	/	/	/	b4	6±0	95.33±2.49	*M. officinalis*
**13**	YK	b1	12±5	50.00±10.80	*C. dactylon*	b1	11±0	76.67±4.92	*C. canadensis*
		b2	13±2	65.00±18.71	*C. dactylon*	b2	6±1	98.67±0.47	*P. arundinacea, C. canadensis*
		b3	7±1	66.67±9.43	*C. dactylon*	b3	9±1	91.00±2.83	*C. dactylon*
**14**	GYT	b1	11±4	72.67±21.08	*P. arundinacea*	b1	9±3	98.33±0.94	*P. arundinacea, C. canadensis*
		b2	6±3	91.67±2.36	*P. distichum, P. australis*	b2	14±2	93.33±3.30	*C. dactylon*
		b3	11±2	90.00±0.00	*C. dactylon*	/	/	/	/

T, transect; YPT, Yangpitan; LHH, Lihuahu; JJZ, Jiangjiazhou; NH, Nanhu; HJZ, Hujiazhou; SBC, Shanbiancun; WMD, Wumingdao; OM, Oumiao; YC, Yicheng; ZX, Zhongxiang; SPZ, Shipaizhen; ZK, Zekou; YK, Yuekou; GYT, Guanyintang.
